# Testing a Digital Health App for Patients With Alcohol-Associated Liver Disease: Mixed Methods Usability Study

**DOI:** 10.2196/47404

**Published:** 2023-11-15

**Authors:** Linda S Park, Rachel Kornfield, Mihret Yezihalem, Andrew Quanbeck, Jessica Mellinger, Margarita German

**Affiliations:** 1 Department of Family Medicine and Community Health School of Medicine and Public Health University of Wisconsin-Madison Madison, WI United States; 2 Preventive Medicine (Behavioral Medicine) Feinberg School of Medicine Northwestern University Evanston, IL United States; 3 University of Wisconsin-Madison Madison, WI United States; 4 Institute for Healthcare Policy and Innovation University of Michigan Ann Arbor, MI United States; 5 Department of Medicine, Division of Gastroenterology and Hepatology School of Medicine and Public Health University of Wisconsin-Madison Madison, WI United States

**Keywords:** alcohol-associated liver disease, ALD, alcohol use disorder, AUD, mobile health, mHealth, digital tools, alcohol cessation, usability test, mobile phone

## Abstract

**Background:**

Alcohol-associated liver disease (ALD) is increasingly common and associated with serious and costly health consequences. Cessation of drinking can improve ALD morbidity and mortality; however, support for cessation is not routinely offered to those diagnosed with ALD, and continued drinking or resumption of drinking after diagnosis is common. Mobile health (mHealth) has the potential to offer convenient and scalable support for alcohol cessation to those diagnosed with ALD, but mHealth interventions for alcohol cessation have not been designed for or evaluated in a population with ALD.

**Objective:**

This study aims to understand how individuals with ALD would perceive and use an mHealth tool for alcohol cessation and to gather their perspectives on potential refinements to the tool that would allow it to better meet their needs.

**Methods:**

We interviewed 11 individuals who attended clinic visits related to their ALD to elicit their needs related to support for alcohol cessation and views on how mHealth could be applied. After completing initial interviews (pre), participants were provided with access to an mHealth app designed for alcohol cessation, which they used for 1 month. Afterward, they were interviewed again (post) to give feedback on their experiences, including aspects of the app that met their needs and potential refinements. We applied a mixed methods approach, including a qualitative analysis to identify major themes from the interview transcripts and descriptive analyses of use of the app over 1 month.

**Results:**

First, we found that a diagnosis of ALD is perceived as a motivator to quit drinking but that patients had difficulty processing the overwhelming amount of information about ALD they received and finding resources for cessation of alcohol use. Second, we found that the app was perceived as usable and useful for supporting drinking recovery, with patients responding favorably to the self-tracking and motivational components of the app. Finally, patients identified areas in which the app could be adapted to meet the needs of patients with ALD, such as providing information on the medical implications of an ALD diagnosis and how to care for their liver as well as connecting individuals with ALD to one another via a peer-to-peer support forum. Rates of app use were high and sustained across the entire study, with participants using the app a little more than half the days during the study on average and with 100% (11/11) of participants logging in each week.

**Conclusions:**

Our results highlight the need for convenient access to resources for alcohol cessation after ALD diagnosis and support the potential of an mHealth approach to integrate recovery support into care for ALD. Our findings also highlight the ways the alcohol cessation app should be modified to address ALD-specific concerns.

## Introduction

The rising rate of alcohol use disorder (AUD) has increased the burden of alcohol-associated liver disease (ALD) [1-3]. The prevalence rate of alcohol-associated cirrhosis alone saw a 43% increase from 2009 to 2015. Among specific demographic groups, those aged >45 years saw a 46% increase, women experienced a 50% rise, and men saw a 30% increase during this period [2]. The COVID-19 pandemic has led to increased rates of alcohol use and has therefore been detrimental to patients with ALD in combination with AUD [4,5]. Siloing of patient care has meant that patients with ALD seen in general hepatology clinics would need to separately seek and coordinate care with addiction medicine to manage their AUD. Rates of referral to AUD treatment for actively drinking patients with ALD from hepatology clinicians are suboptimal, and AUD treatment recommendations in hepatology clinics do not always align with the established guidelines [6]. Only 14.6% of people with ALD receive alcohol treatment [7].

Alcohol abstinence is the only intervention shown to substantially reduce ALD morbidity and mortality [8]. Treatment for achieving abstinence may include a combination of psychological and behavioral approaches (eg, motivational enhancement therapy, cognitive behavioral therapy, and motivational interviewing), self-help (eg, Alcoholics Anonymous [AA]), and pharmacotherapy [9]. However, very few alcohol-focused interventions have been developed and tested in populations with ALD [10,11]. Patients with ALD are also routinely omitted from AUD treatment efficacy studies because of their high mortality and relatively complicated medical condition [12]. Although well established for reducing alcohol use, pharmacotherapies are less often applied in populations with ALD because of concerns about liver metabolism and possible toxicity and the lack of provider education on how to safely prescribe these medications in patients with advanced liver disease [6,13,14]. Interventions that leverage digital technologies are also increasingly available for reducing drinking, supporting a range of strategies, including self-monitoring, goal setting, contingency management, access to therapy modules and psychoeducation, and peer support [15-18]. However, with some exceptions [19], digital interventions have not been studied in populations with ALD [20].

Typically, a major barrier to delivering ALD care to this patient population is that the management of AUD and ALD is siloed between addiction medicine specialists for treatment of AUD and hepatology specialists for treatment of ALD. Integrated AUD and ALD care is a relatively novel way of managing these 2 disorders by having all providers colocated and comanaging the disorders together in 1 multidisciplinary clinic. The few controlled trials that have been conducted in this patient population also show that integrated AUD and ALD care improves the rates of alcohol cessation and sustained abstinence [11].

Patients with ALD also experience additional barriers, both attitudinal and logistical, that prevent their ability to engage in AUD treatment [6,21]. One major barrier is the ability to travel to receive AUD treatment because of the geographic dispersion of patients with ALD. The morbidity of patients with ALD can also compromise their ability to travel [22]. Mobile health (mHealth) apps for alcohol cessation may provide a way to overcome barriers to alcohol use treatment among patients with ALD, as they do not require navigating geographic distances and logistics to access treatment, thereby increasing engagement with alcohol treatment and improving outcomes. Integrating AUD treatment may be a key component of care for patients with ALD because liver disease is a strong motivator for change and can be a key clinical leverage point for AUD treatment engagement [21].

The purpose of this usability test was to establish the feasibility of patients with ALD using an evidence-based mHealth app called Tula (Sanskrit word for *balance*) as part of their ALD care. Tula integrates a number of support strategies for cessation of substance use, including on-demand guidance on cessation strategies, and supports functions such as goal setting and self-tracking. Versions of this mHealth app were found to be effective in multiple randomized clinical trials for patients at risk for severe AUD and for addiction recovery and relapse prevention, including in other substance use disorders [23-27]. Data from this pilot study were gathered to understand how patients use and perceive the app and its usability, not the effectiveness of the app.

Because of the high morbidity and mortality associated with ongoing alcohol use, evidence-based options to connect patients with ALD to some type of AUD treatment while in the hepatology clinic are urgently needed. We address an important gap in ALD patient care—the lack of AUD treatment—and explore whether patients perceive that an AUD treatment app has the potential to support them in managing their alcohol use. Providing an evidence-based mHealth app to patients with ALD could, if proven, set a new standard of care and provide clinicians caring for these patients with an effective means of connecting these patients to life-saving treatment.

## Methods

### Overview

We conducted a mixed methods usability test to assess the feasibility of patients with ALD using an mHealth app in a large Midwestern health care system. Participants were patients at either a general hepatology clinic or a multidisciplinary clinic for patients with ALD and AUD, which combines hepatology care with colocated mental health and AUD treatment [28]. A purposive sampling strategy was used to recruit eligible participants who were identified by their hepatologists during clinic visits [29]. The research staff attended the clinic once a week over a 6-week window to connect with participants who were interested in the pilot study. Refer to Textbox 1 for the inclusion and exclusion criteria. The final sample size was reached once thematic saturation was achieved, meaning that new responses failed to uncover new themes in the data [30]. Each participant could receive a total of US $100 in appreciation of their time.

**Figure 1:**
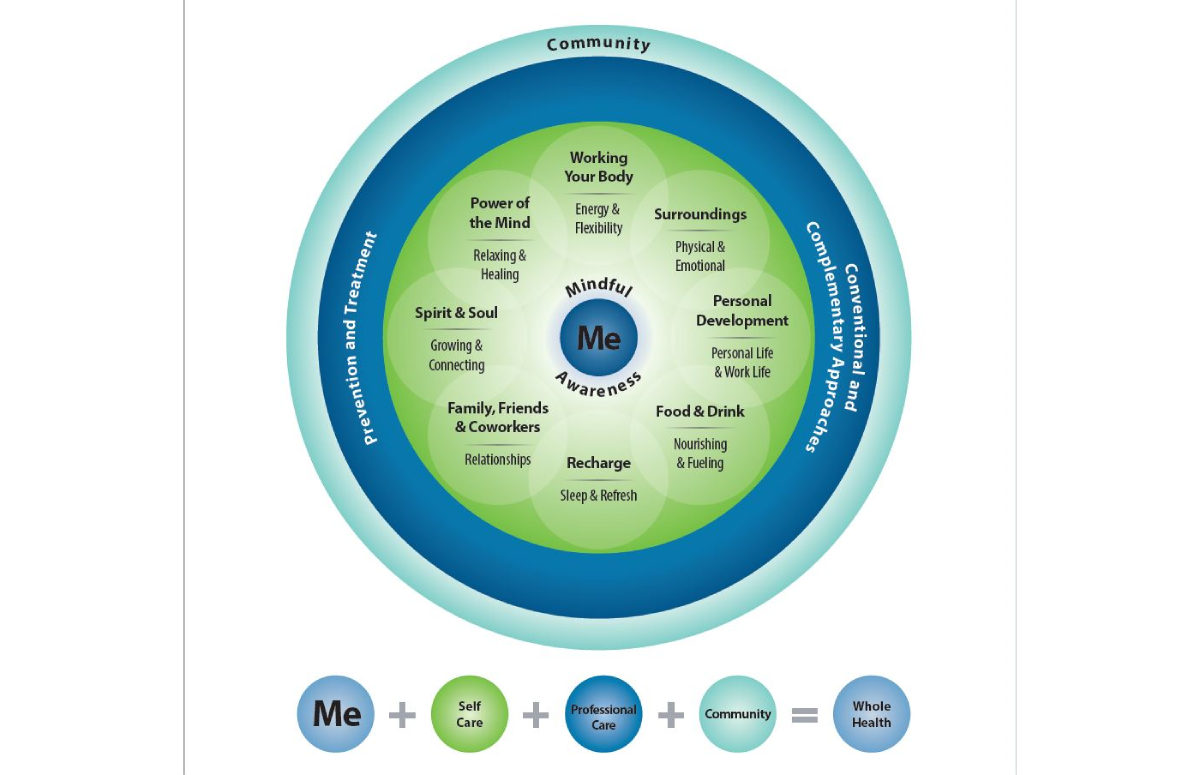
Whole Health Model.

The app is oriented around the Whole Health Model, which provides a holistic model of health based on the principles of integrative medicine [31] (Figure 1).

The model reframes conversations between patients and clinicians from “What is the matter with me?” to “What matters to me?” and encourages patients to envision their overall health along the overall dimensions of well-being, which include the following: power of the mind; moving the body; surroundings; personal development; food and drink; recharge; family, friends, and coworkers; and spirit and soul. The Whole Health Model of care was implemented and evaluated across 18 sites in the US Veteran’s Administration and demonstrated improvements in opioid use, perceptions of care, engagement in health care and self-care, life meaning and purpose, and perceived stress compared with conventional care [32]. This model was used for this study because it is foundational to the model of integrative medicine used at the pilot site and thus fits well with the organizational implementation context.

The theoretical basis of the app is self-determination theory, which holds that helping people meet 3 basic needs improves their adaptive functioning: being perceived as competent, feeling related to others, and feeling internally motivated and uncoerced in one’s actions [33]. Self-determination theory has served as the theoretical basis for various previous versions of the app, upon which our current app is built [23,34]. Prior evidence suggests that its 3 constructs could be causal mechanisms affecting behavioral targets, and the theory is broad enough to explain a complex, multifaceted intervention such as the one we pilot-tested [23,33-35]. The app includes functions such as motivational quotes, informational pages about physical and mental health, tools for journaling with prompts, weekly surveys to track alcohol use, and strategies for alcohol cessation.

**Inclusion and exclusion criteria.**

**Inclusion criteria**

- Age ≥18 y
- Diagnosis of alcohol-associated liver disease and alcohol use disorder
- Alcohol use in the last 6 mo
- Has a smartphone
- Can read and write in English

**Exclusion criteria**

- Needs a liver transplant
- Diagnosis of severe cognitive impairment
- Diagnosis of severe mental illness

### Ethical Considerations

The Health Sciences Institutional Review Board determined that our activities constituted quality improvement and not a human subjects research project. Although this project was not considered “research,” in lieu of an informed consent, we provided every potential participant with a detailed study summary for their review. The research staff were available to answer questions, after which participants provided verbal consent.

### Data Collection

A secure Zoom (version 5.11.3; Zoom Video Communications) interview was scheduled at a date and time convenient to patients interested in participating. All interviews were conducted between August and November 2022. Interview times ranged from 30 to 60 minutes. A total of 20 eligible patients were invited to participate and 11 (55%) were enrolled. Data were collected via semistructured interviews, both before and after participants use of the app for 30 days. Each participant received their incentives directly through Tula as a downloadable Amazon gift card as they completed each segment of the study. Participants could receive US $25 per interview (pre and post), US $10 per weekly survey completed, and a US $10 bonus for completing all segments of the study, totaling up to US $100.

During the preinterview stage, we reviewed the study in detail and answered any questions that participants had; explained the incentives; and gathered demographic information, AUD treatment and support history, and barriers to AUD treatment. We also inquired if the patients had used other mHealth apps for recovery, what their previous experiences were like, and what they hoped to gain from using this smartphone app.

Next, the participants downloaded the app on their smartphones. They were provided with an overview of how to use the app, including guidance on how to complete weekly surveys based on the Brief Addiction Monitor. The weekly survey included questions assessing the extent to which 10 protective and risk factors emerged in their recovery over the past week (eg, sleep, mood, urges to drink, confidence, and relationships), with each response measured on a 7-point Likert scale. Participants completed 4 weekly surveys over a 30-day window, which were recorded in a tracker feature of the app. Throughout the month-long study period, all actions within the app were automatically recorded in time stamped use logs.

After 30 days, we conducted a postinterview to obtain participants’ reflections on their experience with the app and ideas they had for refinement. Participants described the features of Tula that they used the most and least and why. We inquired about any barriers to using the app and whether they would continue using an app such as Tula. We also probed about any additional features or adaptations to the app that might help better meet their needs, including the appropriate balance of content focused on alcohol cessation versus liver disease. Owing to the small number of participants in this pilot study, a peer-to-peer discussion forum was not made available in the app for their use. However, we asked about their thoughts on including a discussion forum comprising other patients with ALD.

### Data Analysis

Our mixed methods approach included a qualitative analysis of data from the participant interviews (both pre and post) and a descriptive quantitative analysis of data generated as the same participants used Tula over the course of a month, focusing on their levels of engagement and allowing for triangulation of the perspectives expressed in the qualitative study [36].

The semistructured interviews were audio recorded and transcribed verbatim using a Health Insurance Portability and Accountability Act–secure transcription service. The categories identified during the interview process became the preliminary codebook. A total of 2 members of the research team and 1 student research assistant independently coded the transcripts using a thematic analysis approach [37]. Categories and codes were refined and agreed upon by the research team using an iterative process to create a final codebook. We used Dedoose (version 9.0.17; SocioCultural Research Consultants; a cross-platform app for qualitative and mixed methods research) to analyze the data. Discrepancies in coding were resolved through group consensus.

We also presented a descriptive analysis of the use of the Tula app. Our analyses excluded the use of Tula, which involved the log-in page, opening and closing the app, and viewing the home page. We computed whether participants used Tula on each of the 30 days subsequent to their training or enrollment date, the total days they used Tula, total pageviews, and total time spent within the app. The time spent on each page was calculated as the total number of seconds before clicking on the next page or closing the app. We capped our calculation of the time spent on each page at 3 minutes to account for pages that may have been left open after participants had disengaged. This 3-minute cap was a 99 percentile of time spent among the participants with ALD, meaning that 99% of the time spent on any 1 page was within 3 minutes (180 s).

## Results

### Participant Characteristics

We enrolled 11 patients with ALD (6 male participants and 5 female participants). Their age range was 33-62 years, with a mean of 46.9 (SD 9.82) years. The additional demographic characteristics are presented in Table 1. The time from diagnosis of ALD to participating in this study ranged from 3 weeks to 20 years, with the majority learning of their diagnosis within 3 years before participation in this pilot study. The history of past alcohol treatment varied, including no formal treatment, rehabilitation (outpatient and inpatient), counseling, medications, and AA. Most patients reported substantial barriers to treatment, including dislike of available group-based treatment options, finances, and logistics (openings for rehabilitation, paperwork, and scheduling). There were 11 participants in total, of whom 10 (91%) completed 2 interviews (1 before and 1 after using the app). There was 1 participant who completed only the initial interview (ie, 21 total interviews).

**Table 1:** Demographic characteristics (N=11).

		Values
**Biological sex, n (%)**		
	Females	5 (45)
	Males	6 (55)
**Race, n (%)**		
	Asian	1 (9)
	White	9 (82)
	Mixed Hispanic and Irish	1 (9)
Age (y), range		33-62
ALD^a^ diagnosis, range		3 wk to 20 y ago, majority within 3 y
Prior mobile health app use for drinking cessation, n (%)		1 (9)

^a^ALD: alcohol-associated liver disease.

### Qualitative Analysis Results

Three major themes emerged from the qualitative data: Participants' Experience with their ALD Diagnosis, Potential Benefits of an mHealth App for ALD, and Participants' Perspective on Refinements Needed to the App.

#### Participants’ Experience With Diagnosis

Participants recalled their ALD diagnosis as a significant experience that shaped their overall understanding of their health and that increased their motivation to abstain from or reduce alcohol use. We identified 3 subthemes in how participants relayed their experience of a new ALD diagnosis, as described in the *Severity of Health Problems*, *Information Overload*, and *Accessing Support and Treatment* sections.

##### Severity of Health Problems

First, although most participants had previously been aware of the problems caused by alcohol use, the diagnosis clarified the potential for serious consequences or even death. According to participant 1, the diagnosis served as “a serious wake up call.” Several participants directly linked the diagnosis of ALD with increased motivation to manage alcohol use or achieve abstinence. Participant 4 described the following:

It was very important because there [were] heavy duty medical consequences that I paid. So I knew, I knew...it’s do or die for me right now.

The diagnosis also led her to challenge the rationalizations she had used before her diagnosis to support continued drinking, such as telling herself “It’s not really, you know, affecting my life.” Similarly, participant 3 described motivation stemming from the possibility of death:

I definitely don’t, like—I don’t want to leave my kid behind.

##### Information Overload

Despite understanding the gravity of their diagnosis, the second theme was that participants felt overwhelmed and overloaded with information, which led to a lack of clarity about the medical implications of ALD. During diagnosis, participants described challenges in navigating the logistics of the visit while attending to the detailed medical information about ALD and dealing with the impact of the diagnosis. For example, participant 10 described the amount of information he needed to process:

...the doctor that saw me that said, you know, you have Cirrhosis, you know, gave me some instructions: this is what you need to do from the physical side of things, like, you need to change your diet. You need to, you know, drink a cup of coffee. You need to quit drinking...but it’s overwhelming, I mean, it’s like, you know, it all gets thrown at you.

Consequently, some reported a limited or incomplete understanding of ALD. Participant 10 went on to express confusion about the effects on his liver if he were to drink:

I hate to say, but I don’t really understand like, you know, if I were to, I don’t understand what that means for what I have left of my life if that’s the right way to say it, you know? Like, is it so bad that like, tomorrow I'm going to die? Or, you know, a year from now?

Similarly, participant 11 reflected the following:

I think initially after diagnosis, I, I didn’t really understand what was going on and didn’t, I guess, have a very good frame of reference for that.

Lacking understanding of the medical implications of ALD could feed into resumed drinking, as experienced by participant 7, who reported that when he began “feeling better” he assumed “that was kind of behind me, and I just essentially continued on with my bad habits until they caught up with me again.”

##### Accessing Support or Treatment

The third subtheme was the challenges faced in translating new motivation after diagnosis into accessing support or treatment to reduce drinking. Despite feeling a new commitment to address their drinking issues, many individuals were confused about specific steps to take or were unhappy with the treatment and support options available to them. For example, participant 5 described the experience of trying to find help with her drinking as “swimming alone.” Ultimately, she was able to connect with self-help only through a fellow patient who showed her how to join a support group through Zoom:

And I was grateful to meet this woman who had actually been through a lot and had been there before. So she’d walked that walk...But anyway, yeah, without her, I would not have even been exposed to Zoom meetings.

Similarly, after diagnosis, participant 10 felt he lacked a clear sense of where to turn, describing the following:

OK, now I got to figure out how to do that...they’re just giving you information, and not giving you too much direction on how to go about doing that, or at least some examples of what you could do...The quitting drinking, I mean, that’s up to me to figure out.

Similarly, participant 11 reported that although his doctor had recommended counseling to help with his recovery, he was unsure “what specific counseling was needed or what was recommended. There was just different avenues, but there [wasn’t] really anything specific that they could bring to me.”

Collectively, these findings suggest the potential of ALD diagnosis to serve as a turning point in addressing alcohol use. However, they also highlight the potential for this transition point to be undermined by the overwhelming amount of information patients need to absorb about ALD and the lack of specific guidance available to help patients harness their motivation toward effective management of drinking. For example, participant 5 reflected on the appeal of testing Tula:

Sometimes the more information you have, the better you are equipped to handle things, so...I’m excited about that.

Participant 3 highlighted the potential value of having resources always available to support recovery:

You know, if I need it in the middle of the night, I can use it or, at any time of the day. That’s what made me really excited hearing about it.

Thus, participants largely saw the value of an mHealth app and were interested in and eager to try Tula.

#### Benefits of an mHealth App

The benefits of the app were a major theme addressed in the postinterviews. This section includes subthemes in participants’ favorite features of the app as well as any challenges encountered using Tula or modifications needed. Participants were consistently pleased with the functionality of the app, and none of the participants mentioned any major barriers to use. They stated that the main benefits of the app are its *tracking feature*, *motivational quotes*, and *ease of use*.

##### Tracking Feature

In total, 90% (9/10) of the participants stated in the postinterviews that the surveys and tracker feature for drinking had a positive impact on their sobriety. The surveys involved answering weekly questions about protective and risky recovery factors, whereas the tracker could be used on demand to set goals around drinking behavior and monitor drinking behavior or other factors of interest. Participant 11 stated the following:

The tracker was probably the most helpful...it gave me a little bit of a reflection on how my mood was changing from week to week.

Some participants, because they were already abstaining from drinking, did not find much use in tracking their drinking. For example, participant 4 stated the following:

Don’t get me wrong I think [the tracker] is great, it’s just that it didn’t really apply to me...with all my answers having been zero.

However, regardless of their current drinking status, several participants appreciated being able to track their mood and relationship with others. When asked what they thought about the weekly surveys, participant 6 said the following:

...it made me sit and think more about urges and triggers...what may have caused [discomfort] with relationships or activities.

In summary, the participants felt that the surveys and tracker within Tula created a safe space to be honest with themselves, and being able to see their progress throughout their time using the app helped motivate them to stay sober.

##### Motivational Quotes

Participants found the motivational quotes to be empowering and supportive. Every day, a motivational quote popped up on the app called “Thought of the Day,” which included quotes from a variety of authors. In total, 50% (5/10) of the participants explicitly stated that “Thought of the Day” was their favorite feature of the app. Participant 4 described this feature as “very inspiring...because it’s so positive, and it makes me think of different ways to maintain my outlook and my health.” An additional daily prompt asked participants to pause and reflect on, “What are you grateful for today.” Participant 10 described how integrating gratitude into their daily routine helped them gain perspective on their recovery:

It takes me back a little bit to where my head was at...a month ago versus where it is now...the things I was grateful for then. Some of them are the same and some of them may have changed a little bit, but it’s interesting to see.

Having the motivational quotes and daily gratitude reminders were a key aspect of keeping participants engaged and consistently using Tula.

##### Ease of Use

Ease of using the app was discussed favorably by 40% (4/10) of the participants in the postinterviews. These participants generally described the app as intuitive. For example, participant 2 stated the following:

I’m a middle-aged woman. I’m not super savvy on the technology and the apps...but I found [Tula] very easy for me to navigate and easy for me to utilize.

Participants especially appreciated having curated information and resources organized into categories. Participant 2 stated the following:

I mean, I can find it online, but then I’d have to go to all these different websites and pick and choose...Whereas with Tula, I was able to just go to that one location and...everything was there.

Furthermore, participants liked the self-directed aspect of the app because of its comprehensiveness and the ability to access it at any time. For example, participant 3 stated the following:

You learn a lot and it helps you be able to work on your own...You can do it on your own time and [at] your own pace, and just find what works for you.

When asked about the benefits of Tula, the participants most frequently stated its tracking feature, motivational components, and ease of use. Being able to learn at their own pace allowed participants to tailor their Tula experience to best fit their individual needs.

#### Improvements Needed to the App

All participants agreed that Tula was helpful but also noted the need for some refinements to make the mHealth app practical and useful for patients with ALD. In the area of improvement needed, 3 subthemes arose: *inclusion of ALD information along with AUD*, *support to improve liver health*, and *an option for a peer-to-peer discussion forum*.

##### Inclusion of ALD Information Along With AUD

Of the 10 participants who completed the postinterview, 9 (90%) wanted the inclusion of both alcohol cessation and liver disease information in an mHealth app. This was particularly true for participants who were sober and already felt that they understood the value of sobriety but wanted to understand ALD more deeply. Other participants who struggled more with drinking and felt that the alcohol cessation aspects would be more valuable for their own circumstances still felt that having ALD information would be important. For instance, participant 6 stated the following:

I guess both, but I think the toughest battle, what’s going to save your life is the alcohol part. Yeah, both, I mean both to me is, is a win-win.

Participant 7 similarly stated the following:

Both would be helpful...they kind of go hand in hand, obviously, with the cessation comes better liver health in general, so—kill two birds with one stone.

Some participants felt that information from their provider was not enough for them to understand a complex disease such as ALD. Many admitted that when they received their diagnosis, they did not really understand what was going on. For example, participant 3 explained the following:

I feel like when I was diagnosed, I really didn’t understand, OK, what does that really mean? So like, I had to go online and be like, OK, what’s stage one, what’s stage two, you know? What are all the different stages, kind of like, what are your options? You know, what kind of health issues can arise?

During the preinterview, participants expressed that they had experienced information overload at the time of their diagnosis; however, after using the app for 1 month, they expressed during the postinterview that having information available on the app might even help them ask better questions to their providers. Overall, participants felt that knowing preventive measures or understanding the trajectory of the disease would help them mentally and physically.

##### Support to Improve Liver Health

Although participants appreciated the whole health perspective of the Tula app, they felt that as patients living with ALD, they wanted information on how to take care of their liver and possibly improve their liver health. Of the 10 participants, 7 (70%) stated that they would like to see more information on nutrition and how it affects their body, especially because many patients with ALD need to be on a low-sodium diet. Participant 1’s statement summarized many participants’ sentiment regarding their liver health:

I guess more so nutrition-wise. You know, there is nutritional facts on there, but maybe having some specific type of diets. I know like I’m supposed to be on a low sodium diet because of my liver. You know, and a lot of the app just kind of goes into more so healthy foods, this and that. And it’s kind of a generalized thing. It’s not really specific.

They wanted to see more information on healthy eating, specifically for better liver function and long-term liver health. Participant 2 stated the following:

Yeah. Nutrition, food, you know, foods that are especially good for liver health would be really beneficial. I mean, we tend to eat a very healthy diet anyway. But you know, if there’s foods that are specifically better for liver function and so forth. We’ll definitely be interested in that.

With the strong motivation for change after receiving their ALD diagnosis, participant 9 expressed the following:

More long-term stuff is interesting ‘cause I’m hoping to stick around for a while, so I guess you know, what options are for long-term liver health is kind of a big thing.

The overall consensus was that an mHealth app could be helpful for learning how to take care of their liver or improve their liver health in addition to learning alcohol cessation strategies.

##### Option for a Peer-to-Peer Discussion Forum

Although the participants of this usability test did not have access to a discussion forum, they were asked about their thoughts on having one available. All but 1 (90%) of the 10 participants (postinterview) liked the idea of having a discussion forum, particularly once they realized that it was not something that was scheduled or face-to-face and was anonymous. Several participants stated that the pressure of sharing their experiences in a face-to-face group setting was daunting; therefore, having the option of web-based, text-based discussions would be appreciated. For instance, participant 6 stated the following:

I love it...Yeah I think, especially in this day and age, you know, like I found it really hard for myself to, and very uncomfortable for me going to like an AA meeting or something like that because, you know, I really don’t want my face out there too much.

Participants connected to the idea of a discussion forum as if it was a support group to help them manage or understand their ALD better. Being able to share similar experiences was welcomed. Participant 4 said the following:

I would be completely for that. I love support groups, and the more help I can get, the better. There’s no such thing as too much help I don’t think.

They also liked the idea of being able to post content or have conversations with others who share similar experiences on their own time frame. Participant 3 shared the following:

That way you could get, you know, exchange tips, exchange stories. Again, it’s almost like going to a group, but not like having to physically leave your house, or you can respond to the discussion group when you have time, or when you’re ready. I think that’d be really cool.

Participants appreciated the idea of being able to have peer-to-peer discussions with others whether as an information source or sharing their experiences with each other without having to travel, schedule a time, or be physically present as in face-to-face meetings (AA) or group therapy.

### Quantitative Descriptive Analysis

#### App Use

**Figure 2:**
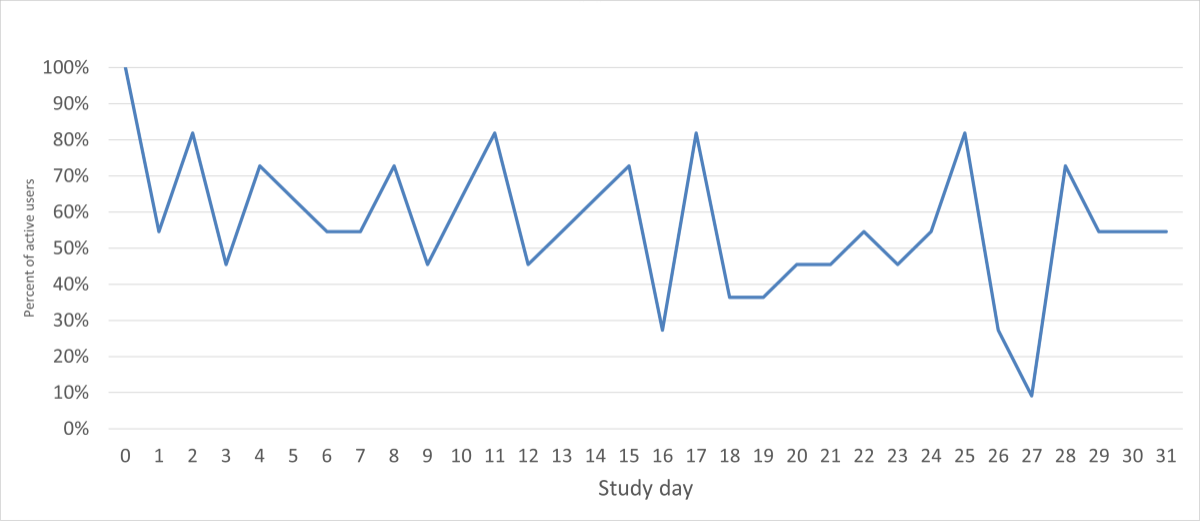
Percentage of participants active on the Tula app on each day of the study.

Participants spent an average of 31.9 minutes using Tula on the day they enrolled in the study and were oriented to the app. For the 30 subsequent days (relative to each participant’s enrollment date), participants engaged with Tula beyond the home page on an average of 17.1 days, registering an average of 209.4 total page views and spending an average of 40.5 minutes in the app in total. On any given day, the percentage of participants active on the system was approximately 55% (6/11), with slightly declining rates of daily use over time, as illustrated in Figure 2. Weekly engagement rates were 100% (11/11), meaning that all 11 participants opened the app and went beyond the home page to access Tula’s services during each week of the study. Participant completion of the weekly surveys was also 100% (11/11), meaning that all participants completed the 4 weekly surveys distributed through the app.

## Discussion

### Principal Findings

Our pilot study is among the first to apply a usability test for an mHealth app to help patients with ALD and AUD. Interested patients with ALD used Tula, an evidence-based mHealth app based on the Whole Health Model to support alcohol use reduction with the goal of cessation, for 1 month [23,38]. We found that patients with ALD in this study perceived the mHealth app as both easy to use and useful in helping them deal with alcohol cessation, especially through the self-tracking and motivational features of the app. As perceiving a tool as easy to use and useful are key predictors of technology adoption [39], these findings highlight the promise of an mHealth approach for ALD. Participants’ endorsements of the app’s self-tracking feature and daily quotes also correspond to the prior literature. Prompting individuals to record health behaviors is theorized to build self-regulatory capacity and has been associated with the effectiveness of interventions that target excessive drinking [40]. Many individuals are motivated to use digital interventions to conveniently self-track and self-monitor drinking [41,42], and tracking features can help individuals build self-awareness and manage their behaviors to align with their goals [43,44]. User-centered design studies have also surfaced the value of daily quotes and other brief encouraging messages as a source of novelty and motivation, supporting sustained engagement [45,46]. We found that, on average, participants engaged with the app on more than half of the study days and that all 11 participants used the app each week of the study, which exceeded weekly app use rates in prior research conducted in a population of 269 patients with a range of substance use disorders [47]. Participants also made suggestions regarding app modifications, such as the addition of information to help them understand their ALD diagnosis and how to care for their liver. Another feature that they endorsed was the ability to connect with other patients with ALD for peer-to-peer support.

Although ALD diagnosis was a potential turning point at which patients with ALD felt increasing motivation and urgency to address their alcohol use, participants were overwhelmed by all the information they needed to absorb regarding managing their ALD. Participants appreciated having curated information and resources organized into categories and the ability to access whatever topics they were interested in at any time. This access to on-demand information may help patients to increase their health literacy, which is defined by the World Health Organization as the ability to achieve “a level of knowledge, personal skills, and confidence to take action to personal and community health by changing personal lifestyles and living conditions” [38]. Kaps et al [48] found that patients with liver cirrhosis are at a higher risk of lower health literacy. They also found that “poor liver function, as defined by higher Model for End-Stage Liver Disease scores, independently correlated with poorer ability to find good health information and manage one’s own health, and a feeling of not having sufficient information to manage health” [48]. Their findings were consistent with those of other studies investigating chronic diseases [49,50]. Studies also found that the health literacy of people with alcohol or substance use disorders is low [51]. Therefore, an increase in health literacy around ALD and AUD combined could not only help this patient population take care of their liver and manage their alcohol consumption but also potentially extend into improved health literacy overall. Future research could examine the role of mHealth apps such as Tula in health literacy and the transfer of improved health literacy to other mediums and health conditions.

Whether facing a new diagnosis or worsening medical symptoms, participants in this pilot study were motivated to stop drinking once they understood that alcohol cessation was the only treatment intervention to improve long-term mortality and liver health. Motivation can play a major role in alcohol cessation and engagement with treatment [51,52]. Kelly and Greene [53] found that high levels of motivation can play an important role in predicting abstinence even when individuals have low levels of self-efficacy for cessation. However, although participants reported that the initial impact of their ALD diagnosis was a strong motivator to stop alcohol use, they were eligible for our study because they had consumed alcohol within the last 6 months. Participants acknowledged that they needed help to maintain motivation in their day-to-day life and build skills and confidence. For some, regular motivational quotes and daily gratitude reminders through Tula were key aspects of staying engaged in their recovery. For others, the weekly surveys and tracker feature provided real-time information about progress toward abstinence or attempts to reduce alcohol use, perhaps increasing their self-efficacy regarding alcohol cessation. Our findings suggest that, although the app was well received as a whole, some key features (eg, motivational quotes, daily gratitude, weekly surveys, and tracker feature) may have played an especially important role in keeping participants engaged with the app and possibly extending their motivation beyond the time point of ALD diagnosis.

### Practical Implications of Findings for App Design and Integration in Care

Our findings have practical implications for the design and deployment of digital tools to support patients with ALD. In terms of design, our findings suggest that there are dual needs in this population: (1) to support alcohol cessation and (2) to provide education and guidance regarding ALD. The existing alcohol cessation aspects of Tula were perceived as useful and usable, including daily motivational content and self-tracking features. Although Tula does not contain ALD-specific information or resources, our findings support their integration. Revisions to the app may include information about ALD (eg, prognosis, symptoms, and treatments) and daily care for one’s liver beyond alcohol cessation (eg, diet and exercise). As participants reported that the volume of information received in ALD clinic visits was often overwhelming and difficult to understand, attention may be warranted to break this content into manageable modules that can be consumed in sequence or explored on demand, as well as providing information at the appropriate level of health literacy [52,54]. Given the complexity of ALD and its management, interventions may include an option to “ask an expert,” with questions submitted through the app to a clinical team member who responds asynchronously. Such an option has been integrated within digital health tools for other health conditions to manage questions not addressed within the informational materials [55]. Our findings also support integrating peer-to-peer communication through a discussion forum. Such components have been an engaging and valued aspect of various recovery apps and digital tools, offering on-demand social support [56]. Social support is a strong predictor of managing and coping with both ALD and AUD [57-59]. This support may be especially valuable coming from individuals who share an ALD diagnosis because of the potential to draw on personal experience for emotional and informational support.

Regarding the integration of an mHealth approach into care, our findings suggest that patients are open to using a digital recovery tool when it is offered by a hepatologist as part of an ALD clinic visit. They highlight the benefits of providing this tool soon after ALD diagnosis, as the tool may help patients to continue to consume and process information about ALD after they have returned home, as well as allowing them to channel the increased motivation to reduce drinking that comes from an ALD diagnosis into sustained behavior change. An mHealth approach could allow hepatologists to integrate behavioral health care into ALD treatment in a way that is inexpensive and requires minimal staff support (eg, a brief training during the clinic visit). Although this study examined the independent use of Tula, with no interaction related to Tula between the patient and care team beyond referral to our study, further research may examine whether and how health care teams might be involved to provide ongoing support of Tula use or to access data from Tula to inform more personalized care. Digital health research increasingly seeks to understand the human support that underlies the successful incorporation of mHealth into health care [60,61]. Key roles may include training and orientation to a tool, encouragement and support of tool use, moderating peer-to-peer discussion forums, or integrating data into clinical care [62]. Recognizing the need for behavioral health care in patients with ALD, some recent efforts have been made to establish multidisciplinary ALD clinics, in which a patient sees not only a hepatologist but also addiction specialists [63]. Future work should evaluate whether traditional hepatology clinics or multidisciplinary clinics are more appropriate sites to implement a solution such as Tula. Any integration of data from Tula into health care must also balance the preferences of patients, who may share less openly via the app or disengage if data reported through an app are used in ways that feel punitive (eg, affecting candidacy for liver transplant).

### Limitations

This pilot study has several limitations. First, although we reached theme saturation with 11 patients, the small number of participants makes it difficult to generalize the results to a larger population. The study participants were also predominantly White (9/11, 82%). Although the study participants reflected the demographics of the geographic area served by the clinic, the inclusion of ethnic and racial minority individuals in research is important to ensure that tools that meet the needs of diverse populations and promote health equity are developed [64]. In addition, participants were recruited from 1 hepatology clinic, which brings about 2 other limitations: other clinics in other health care systems may be set up differently and people without access to health care were not considered for the study. All these limitations could be addressed by conducting a larger-scale study and purposefully composing a diverse sample of participants.

### Conclusions

Many indicators since the COVID-19 pandemic suggest that ALD will become a bigger problem in the coming years. Most patients with ALD in the United States are treated in general hepatology clinics, which focus on treating the complications of all liver diseases but often do not provide extensive support for alcohol abstinence. Because of the high morbidity and mortality associated with ongoing alcohol use, evidence-based options to connect patients with ALD to some type of AUD treatment while in the hepatology clinic are urgently needed. Our results highlight the need for convenient access to resources for alcohol cessation after ALD diagnosis and support the potential of an mHealth approach to integrate recovery support into care for ALD. Our findings also emphasize the necessary modifications to the alcohol cessation app to effectively address the specific concerns related to ALD. Providing an evidence-based mHealth app to patients with ALD could, if proven, set a new standard of care and provide clinicians caring for these patients with an effective means of connecting them to life-saving treatment.

## Abbreviations

AA: Alcoholics Anonymous
ALD: alcohol-associated liver disease
AUD: alcohol use disorder
mHealth: mobile health
